# The effects of actuator selection on non-volitional postural responses to torso-based vibrotactile stimulation

**DOI:** 10.1186/1743-0003-10-21

**Published:** 2013-02-13

**Authors:** Beom-Chan Lee, Bernard J Martin, Kathleen H Sienko

**Affiliations:** 1 Department of Mechanical Engineering, University of Michigan, Ann Arbor, MI, USA; 2 Department of Industrial & Operations Engineering, University of Michigan, Ann Arbor, MI, USA; 3 Department of Biomedical Engineering, University of Michigan, Ann Arbor, MI, USA

**Keywords:** Vibrotactile stimulation, Directional response, Tactor type, Proprioception, Balance, Biofeedback, Vibrotactile displays, Sensory augmentation

## Abstract

**Background:**

Torso-based vibrotactile feedback may significantly reduce postural sway in balance-compromised adults during quiet standing or in response to perturbations. However, natural non-volitional postural responses to vibrotactile stimulation applied to the torso remain unknown.

**Methods:**

The primary goal of this study was to determine, for two types of actuators (tactors) and in the absence of instruction, whether vibrotactile stimulation induces a directional postural shift as a function of stimulation location. Eleven healthy young adults (20 – 29 years old) were asked to maintain an upright erect posture with feet hip-width apart and eyes closed. Two types of tactors, Tactaid and C2, which differ in design and stimulation strength, were placed on the skin over the right and left external oblique, internal oblique, and erector spinae muscles in a horizontal plane corresponding approximately to the L4/L5 level. Each tactor of the same type was activated twice randomly for each individual location and twice simultaneously for all locations at a frequency of 250 Hz for a period of 5 s.

**Results:**

Vibration applied over the internal oblique and erector spinae muscle locations induced a postural shift in the direction of the stimulation regardless of the tactor type. For the aforementioned four locations, the root-mean-square (RMS) and power spectral density (PSD) of the body sway in both the A/P and M/L directions were also significantly greater during the vibration than before or after, and were greater for the C2 tactors than for the Tactaid tactors. However, simultaneous activation of all tactors or those over the external oblique muscle locations did not produce significant postural responses regardless of the tactor type.

**Conclusion:**

The results suggest that the use of a torso-based vibrotactile sensory augmentation display should carefully consider the tactor type as well as the instruction of corrective movements. Attractive instructional cues (“move in the direction of the vibration”) are compatible with the observed non-volitional response to stimulation and may facilitate postural adjustments during vibrotactile biofeedback balance applications.

## Background

Tactile displays are human-computer interfaces that use tactation to present information [1]. Various actuation methods (e.g., electromechanical, electromagnetic, piezoelectric crystal, pneumatic actuation) have been designed to present spatial and temporal information, such as object shape, surface texture, movement direction, and emotion [2,3]. Early tactile displays used to present text, graphic shapes, maps, and images to the fingertips typically consisted of arrays of actuators that raised and lowered pins through holes in a flat surface [2,3]. More recent displays consisting of electromechanical units that generate inertial, rotational, or linear vibration have been effective in conveying navigation information to aid individuals with visual impairments [4,5], attitude information or threat warning cues to aircraft pilots [6,7], orientation and proximity information to foot soldiers [8], and directional information to drivers [9,10].

Since 2001, vibrotactile displays have been used in balance-related applications in which cutaneous stimulations provide information about body motion with respect to the gravito-inertial vector in order to inform corrective motor responses [11]. These volitional corrective responses have been associated with decreased postural sway in individuals with vestibular deficits [12-17], older adults [18], and healthy young adults [16-19] during quiet and perturbed stances. To date, the most frequently used vibrotactile displays for balance-related applications have employed an array of either inertial or linear electromechanical actuators (tactors) placed along a belt worn horizontally around the torso [12-19].

Traditionally, repulsive cuing strategies, i.e., users are instructed to move away from the activated tactor, have been used for balance-related applications. Wall et al. [11] used this approach on the basis that vibration may provoke an aversion response similar to the response generated by an individual who encounters an obstacle. Subsequent studies have employed a similar scheme [12-19]. However, the postural adjustment is simply considered to be a volitional response to a warning signal, which may not be congruent with possible kinesthetic information from the stimulated tactile receptors. Previous studies have shown that cutaneous receptors located in the skin around finger, elbow, ankle, and knee joints provide exteroceptive and proprioceptive information [20-23]. Similar to muscle spindles, these receptors both encode movement kinematics and show directional sensitivity [20-23]. We have previously demonstrated the contribution of cutaneous receptors to the spatial representation of the torso and the potential incompatibility between aversive volitional responses and vibration-induced non-volitional postural adjustments in the absence of instruction [24].

For vibrotactile-based balance applications, arrays of commercially available tactors are commonly used to provide vibrotactile instructional cues [18,25,26]. However, the effects of tactor type on non-volitional postural responses to vibrotactile torso stimulations are unknown. One hypothesis is that the strength of the vibration-induced directional postural shifts and/or postural alterations will differ if both the number of tactile receptors recruited as well as the resulting sensory afferent flow differ between each type of stimulation.

The purpose of this study is to investigate the influence of tactor type on the direction and magnitude of postural responses induced by vibrotactile stimulation applied to various locations around the torso in the absence of instruction. The results from this study will inform the selection criteria for tactor type and application locations for torso-based vibrotactile sensory augmentation balance devices. An earlier version of the results has appeared in abstract form [27].

## Methods

### Instrumentation

**Figure 1:**
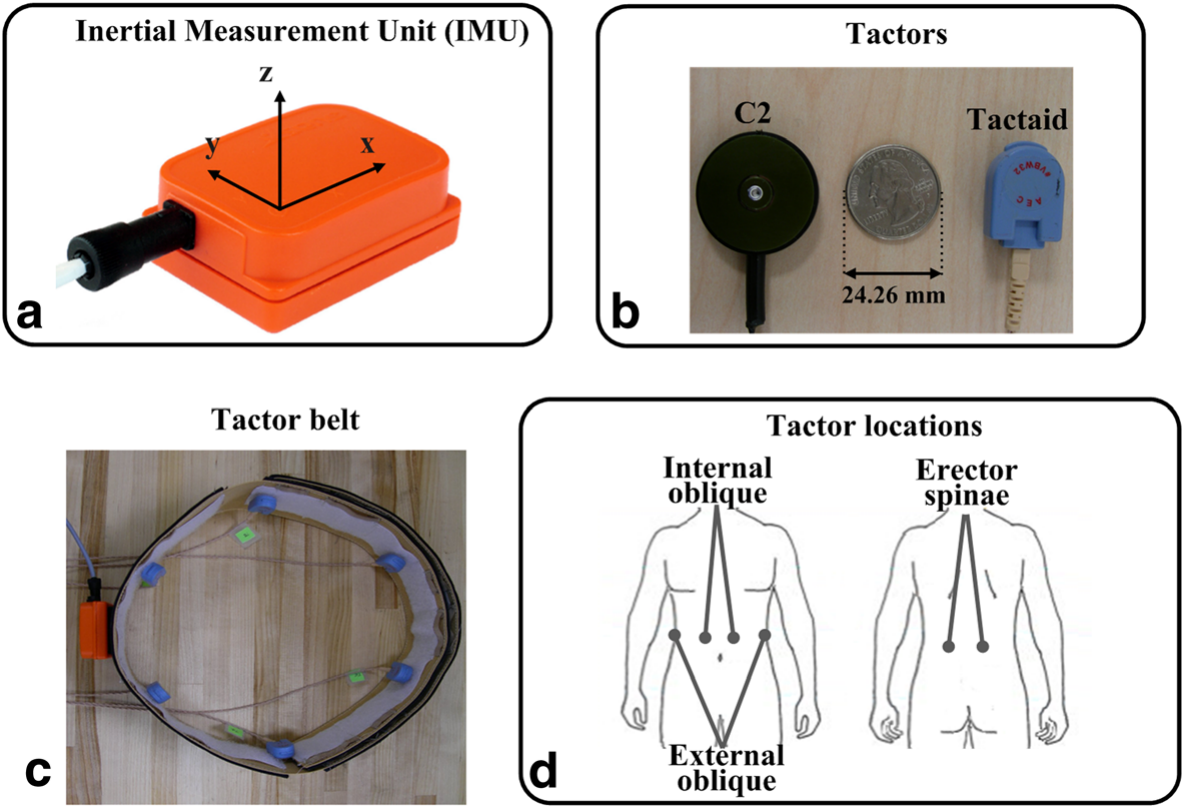
(a) Inertial measurement unit (IMU). (b) C2 and Tactaid tactors. (c) Elastic tactor belt with Tactaid tactors and IMU. (d) Stimulation locations.

Figure 1 illustrates the components of the experimental apparatus, which include a six degree-of-freedom inertial measurement unit (IMU; Xsens Technologies, NL), six C2 tactors, six Tactaid tactors, and an elastic belt. The IMU, placed on the back of the torso at approximately the L3 vertebra level, measured upper body angular displacements, velocities, and accelerations in the anterior-posterior (A/P) and medial-lateral (M/L) directions. These signals were sampled at a rate of 100 Hz. The IMU static accuracy is better than 0.5°, with an angular resolution of 0.05°.

The Tactaid VBW32 tactor, an electromagnetic inertial transducer, consists of a mass suspended on a spring inside a rigid casing. Both the mass and the casing vibrate in a plane normal to the skin when an alternating electromagnetic force is generated [28,29]; the subject feels the vibrations through the casing. The C2 tactor is a voice-coil-type linear actuator that incorporates a moving contactor lightly preloaded against the skin [28,29]. The contactor diameter measures 0.8 cm and it oscillates perpendicularly to the skin, while the surrounding skin area is shielded with a passive housing. Thus, the user only feels the vibration through the contactor. The contact areas of the Tactaid and C2 tactors are 3.74 cm^2^ (square area) and 6.15 cm^2^ (circular area), respectively.

The IMU and the tactors were attached with Velcro to an elastic belt worn around the torso, as shown in Figure 1(c). Six tactors of a single type (e.g., either Tactaid or C2) were placed on the skin over the right and left internal oblique, external oblique, and erector spinae muscles approximately at the level of the iliac crest, which corresponds to the L4/L5 vertebrae level, as shown in Figure 1(d). Tactors were driven by a 250 Hz sinusoidal signal through a customized control circuit to maintain the stimulation within the one-to-one frequency response (cyclic synchronized response) of fast-adapting cutaneous receptors [30,31] and to avoid the response of muscle spindles [32,33].

To compare the relative vibration amplitudes of the two types of tactors, we constructed a measurement apparatus comprising a Polytec OFV-3001 Laser Doppler Vibrometer (LDV) (Plytec Inc.) and simulated skin substrate; adhesive was used as a skin/vibrator interface attachment. The LDV instrument is used to make non-contact measurements of surface vibrations based on interferometry. The beam was focused on the center of the tactor. The voltage output, proportional to the instantaneous displacement of the tactors, was recorded at a rate of 250 kHz. The LDV provided a an output voltage resolution of 1 mm/s/V. The measured voltage signals were integrated to compute the displacement of each tactor type [34]. The measured peak-to-peak displacements of the C2 and Tactaid tactors were approximately 200 μm and 50 μm, respectively.

### Subjects

Eleven healthy young adults (22.9 ± 4.8 yrs, 4 females and 7 males) naïve to the purpose of the experiments participated in this study. Exclusion criteria included any central neurological dysfunction (e.g., stroke, myelopathy, vertigo), functionally significant musculoskeletal dysfunction, neurological disease (e.g., cerebral vascular accident, Parkinson’s disease), use of a walking aid, or a body mass index greater than 30 kg/m^2^. All subjects were instructed not to take medications that could cause drowsiness or dizziness and not to consume alcoholic beverages within 48 hours of the experimental session. Informed consent was obtained from each subject prior to the start of the experimental procedures. The study, which conformed to the Helsinki Declaration, was approved by the University of Michigan Institutional Review Board.

### Procedure

The subjects were asked to stand erect on a firm surface, eyes closed, with their arms held at their sides and their feet hip-width apart at a 15° lateral rotation angle. Foam ear plugs and ear muffs were provided to eliminate environmental noise as well as noise due to tactor activation. No specific instruction was given except to maintain an upright stance. No information was provided regarding tactor type, vibration location, and vibration duration.

All subjects completed two distinct series of trials corresponding to each tactor type. The initial tactor type was randomly assigned to each subject. During the experimental protocol the tactors were activated either individually (referred to as “single location” stimulation condition) or simultaneously (referred to as “all locations” condition). Each trial was composed of consecutive measurement periods that included an initial period of 5 s without stimulation (pre-vibration) followed by 5 s of stimulation (per-vibration) and then followed by 5 s without stimulation (post-vibration). Two trials for each stimulation condition were repeated in a random order for a total of 14 trials for each tactor type per subject (i.e., six “single locations” and one “all locations” trials per tactor type). The subjects were instructed to bend at the waist in both the A/P and M/L directions during each 5 s rest period between trials. At the end of the experiment the subjects were asked to indicate by yes or no whether the vibration intensity from each of the six locations was consistent during the experiment and then to indicate which set of tactors (1^st^ or 2^nd^) generated the stronger vibration.

### Data analysis

MATLAB (The MathWorks, Natick, MA) was used to process the postural sway signals captured by the IMU. Detailed information regarding the data analysis methods was presented in a previous work [24]. Three metrics were defined to quantify the postural responses to vibrotactile stimulation: the postural shift vector (indicating the magnitude and direction of postural shift), the root-mean-square (RMS) of the angular displacements of the A/P and M/L body (sway), and the power spectral density (PSD) of the A/P and M/L body sway.

**Figure 2:**
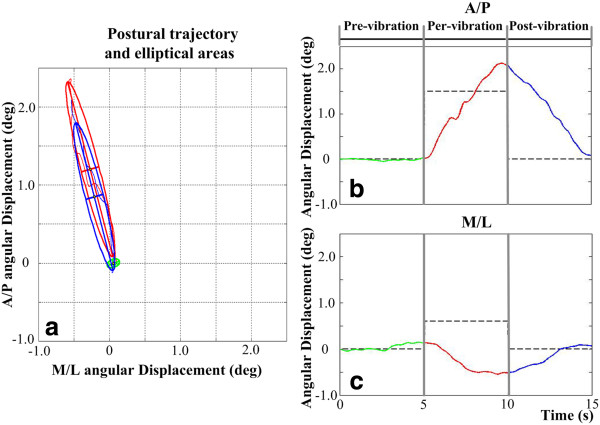
**(a) Illustrative postural trajectories and 95% confidence interval elliptical fits for each vibration period when the tactor was placed over the left internal oblique.** Positive values are defined as movements in the anterior and lateral right directions. (**b**) Illustrative A/P postural trajectories. Positive values are defined as movements in the anterior direction. (**c**) Illustrative M/L postural trajectories. Green, red, and blue lines represent pre-, per-, and post-vibration periods, respectively.

To determine the magnitude and direction of postural responses between the consecutive periods of interest (pre-/per- and per-/post-vibration periods), 95% confidence interval ellipses were fitted to the 2D postural trajectories for each period as illustrated in Figure 2(a). The center of each ellipse was used to calculate the 2D postural shift vector for the pre-, per-, and post-vibration periods. The A/P and M/L RMS values of body sway were computed for the pre-, per-, and post-vibration periods. PSD analysis was used to determine the spectral distribution of power and the dominant frequency of body sway in the A/P and M/L directions. The PSD functions were computed using a discrete Fourier transform (DFT) to decompose the angular displacements of the body into sinusoidal components [35]. The DFT was applied to each 5 s period and computed in the 0.2 – 4.0 Hz frequency range with a resolution Δ*f* = 0.2 Hz. The power in the PSD above 1.0 Hz was less than 13 deg^2^/Hz and no significant difference in the power across the measurement periods (i.e., pre-, per-, and post-vibration) was observed regardless of tactor type. Thus, only PSD magnitudes for frequencies less than 1.0 Hz were considered for data analysis for each tactor type. The magnitudes and directions of the postural shift vectors as well as the RMS and PSD values in both A/P and M/L directions were computed for each subject and each period as a function of the stimulation location. Given that the effect of repetition was not significant, each metric was quantified by the average over two trials.

A three-way analysis of variance (ANOVA) was conducted to determine the main effects of tactor type (C2, Tactaid), vibration location (six “single locations” and one “all locations” conditions), and period (pre-, per-, and post-vibration) for each dependent variable (e.g., magnitude, direction, A/P RMS, M/L RMS, A/P PSD, and M/L PSD). To determine which factors influenced the main and interaction effects, post-hoc tests (Tukey Honestly Significant Differences – HSD – for multiple comparisons) were also conducted. The level of significance was set at *p* < 0.05. To ensure the assumptions of normality and constant variance of residuals, both the A/P and M/L RMS sway values were logarithmically transformed.

## Results

**Figure 3:**
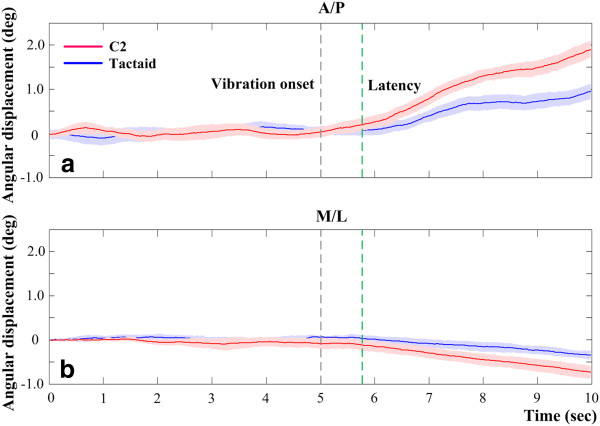
**(a) Average A/P postural trajectories.** Positive values correspond to movements in the anterior direction. (**b**) Average M/L postural trajectories. Positive values correspond to movements in the lateral right direction. Red and blue lines represent average postural trajectories for the C2 and Tactaid tactors, respectively. Shaded areas indicate standard error of the corresponding average postural trajectories.

Figure 2 illustrates the representative results for a single subject when vibration was applied to the skin over the left internal oblique muscle location. Posture shifted in the direction of the vibrotactile stimulation during the per-vibration period. A post-effect, indicated by a shift reversal, was also observed when the vibrotactile stimulation ceased. Figure 3 shows the mean postural trajectories across all subjects in both the A/P and M/L directions during the pre- and per-vibration periods when vibration was applied to the skin over the left internal oblique muscle location. The average latency of vibration-induced postural shifts was 800 ms after the onset of vibration for both tactor types when vibration was applied over the left internal oblique location. This latency was not statistically different (*p* > 0.05) for the right and left internal oblique and erector spinae locations for both tactor types. The pre-, per-, and post-vibration periods are subsequently referred to as before, during, and after the vibration, respectively.

### Magnitude and direction of postural shift vectors

**Figure 4:**
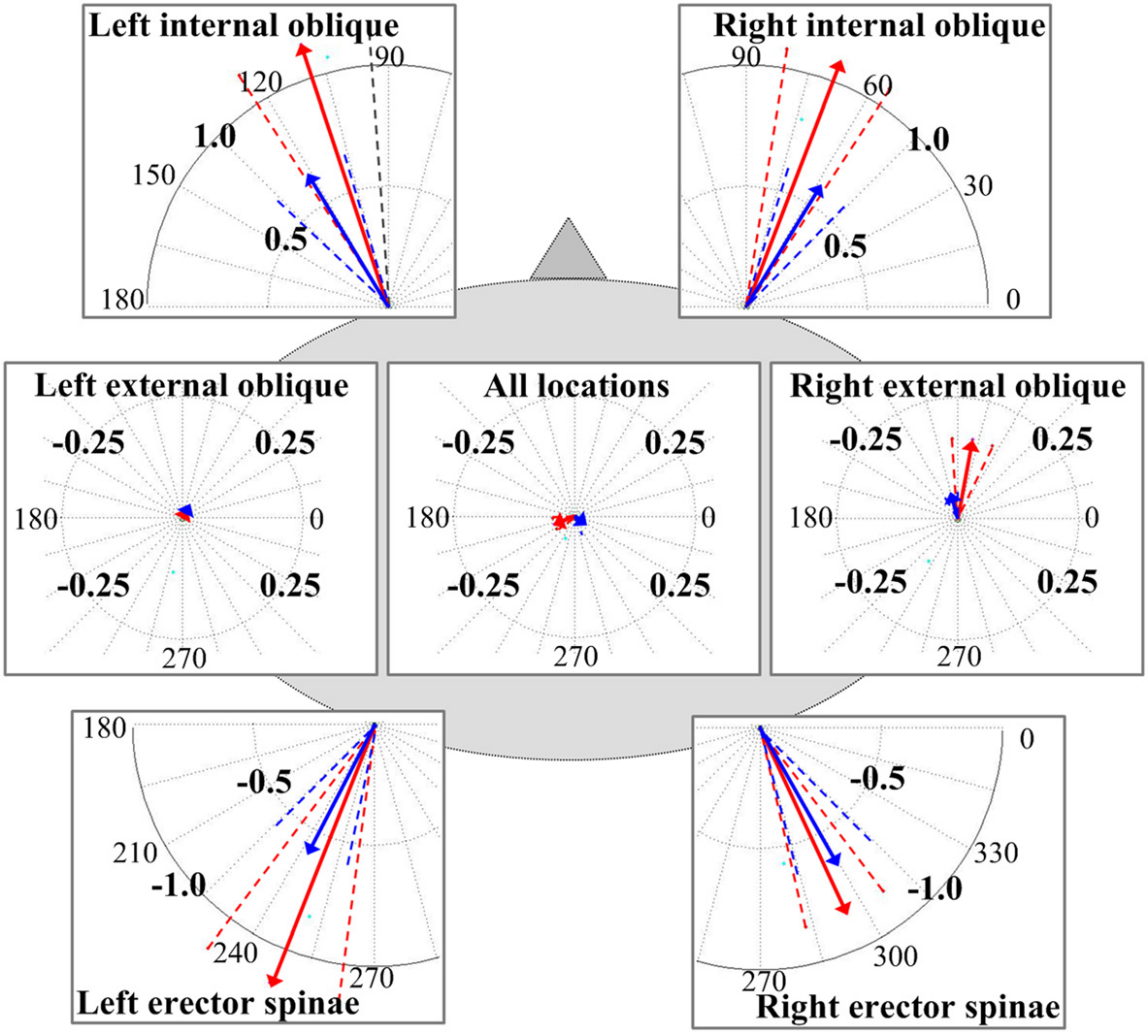
**Average postural shift vectors during vibration as a function of tactor location.** Red and blue vectors correspond to shifts induced with the C2 and Tactaid tactors, respectively. Dashed lines indicate standard error of the corresponding mean vector direction.

Figure 4 shows the postural shift vectors during the vibration period as a function of the stimulation condition and tactor type. The center of the pre-vibration period ellipse was considered to be the origin for the subsequent postural shift analyses.

Table 1 shows that the ANOVA applied to the *magnitude* of the postural shift vectors indicated that the main effects of tactor type, location, and period, and the “tactor type × location” and “location × period” interactions were significant. Post-hoc analysis found that the magnitude of the postural shift vectors was greater during than before or after vibration for both tactors (Tactaid: *p* < 0.01 and C2: *p* < 0.02, Tukey HSD) when vibration was individually applied over the internal oblique and erector spinae locations, but the magnitudes of the postural shift vectors during vibration were not significantly different (*p* > 0.05) between the aforementioned four locations. Table 1 also shows that the ANOVA applied to the *direction* of the postural shift vectors indicated that the main effects of tactor type, location, and period, and the “tactor type × location” and “location × period” interactions were significant. When vibration was individually applied over the right and left internal oblique locations, the subjects exhibited a postural shift in the forward right and forward left directions, respectively, regardless of tactor type. In addition, when vibration was applied over the right and left erector spinae, the body posture shifted in the backward right and backward left directions, respectively. Upon cessation of the vibration, the body posture shifted in the direction opposite to the postural shift observed during vibration, regardless of tactor type. Furthermore, both the magnitude and the direction of the postural shift vectors were not significantly different (*p* > 0.05) across measurement periods when vibration was applied over the external obliques or at all locations regardless of tactor type.

**Table 1:** **Statistically significant results of the dependent variables** (**i**.**e**., **tactor type** (**T**), **location** (**L**), **and period** (**P**)) **and their interactions**

**Dependent variable**	**Effects**	**DF**	**F Value**	**Pr > F**
Postural shift magnitude	T	1, 420	15.71	< 0.0001
	L	6, 420	25.14	< 0.0001
	P	2, 420	62.36	< 0.0001
	T x L	6, 420	4.21	< 0.0001
	L x P	12, 420	4.20	< 0.0001
Postural shift direction	T	1, 420	13.38	< 0.0001
	L	6, 420	7.86	< 0.0001
	P	2, 420	57.54	< 0.0001
	T x L	6, 420	3.24	0.010
	L x P	12, 420	3.86	< 0.0001
A/P RMS	T	1, 420	13.49	< 0.0001
	L	6, 420	51.32	< 0.0001
	P	2, 420	70.37	< 0.0001
	T x L	6, 420	2.59	0.018
	L x P	12, 420	1.91	0.032
M/L RMS	T	1, 420	10.87	0.001
	L	6, 420	60.63	< 0.0001
	P	2, 420	55.806	< 0.0001
	T x L	6, 420	2.41	0.026
	L x P	12, 420	4.99	< 0.0001
A/P PSD	T	1, 420	7.23	0.015
	L	6, 420	52.37	< 0.0001
	P	2, 420	112.04	< 0.0001
	T x L	6, 420	2.29	0.034
	L x P	12, 420	11.63	< 0.0001
M/L PSD	T	1, 420	5.47	0.02
	L	6, 420	78.54	< 0.0001
	P	2, 420	139.86	< 0.0001
	T x L	6, 420	2.45	0.024
	L x P	12, 420	23.77	< 0.0001

**Figure 5:**
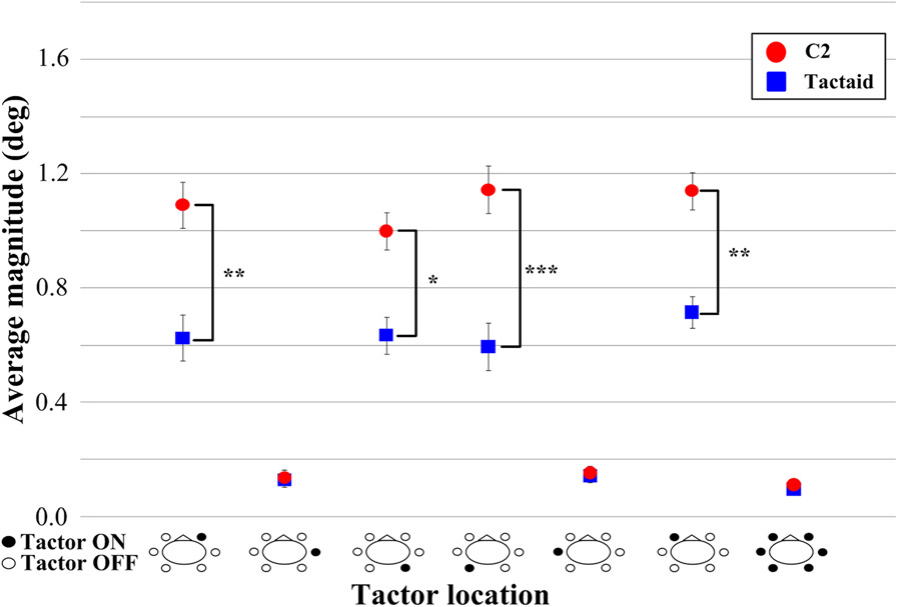
**Average magnitude of the postural shift vector for the C2** (**circles**) **and Tactaid** (**squares**) **tactors during vibration as a function of tactor location.** Error bars indicate standard error of the mean (*p < 0.05, **p < 0.01, ***p < 0.0001). Bird’s-eye view drawings illustrate vibration locations.

Figure 5 shows the average magnitude of the postural shift vectors during vibration as a function of tactor location. The magnitudes of the postural shift vectors were significantly greater with C2 tactors than with Tactaid tactors when vibration was applied over the internal oblique and erector spinae locations. For these four locations, the average magnitude of the vibration-induced postural shift was on the order of 1.2° for C2 and 0.7° for Tactaid. The average time to reach the value corresponding to the center of the per-vibration ellipse was approximately 3 s when vibration was applied over the internal oblique and erector spinae locations regardless of tactor type. Figure 5 also shows that regardless of tactor type, the magnitudes of the postural shift vectors were not significantly different (*p* > 0.05) across measurement periods when vibration was applied over the external oblique locations or over all locations simultaneously.

### RMS sway

Table 1 shows that the ANOVA applied to the RMS sway indicated that the main effects of tactor type, location, and period, and the “tactor type × location” and “location × period” interactions were significant for both A/P and M/L directions. Post-hoc analysis found that both the A/P and M/L RMS sway values were significantly greater (Tactaid: *p* < 0.02 and C2: *p* < 0.014, Tukey HSD) during and after than before vibration when stimulation was applied over the internal oblique and erector spinae locations. There were no differences (*p* > 0.05) among the A/P and M/L RMS sway values during and after the vibration period across the internal oblique and erector spinae locations regardless of tactor type. Post-hoc analysis also found that the A/P and M/L RMS sway values before vibration were similar (*p* > 0.05) across the six single locations regardless of tactor type. Further, the A/P and M/L RMS sway values during vibration were similar (*p* > 0.05) for the internal oblique and erector spinae locations regardless of tactor type. However, changes in both the A/P and M/L RMS sway values were negligible when vibration was applied over the external obliques or over all locations regardless of tactor type.

**Figure 6:**
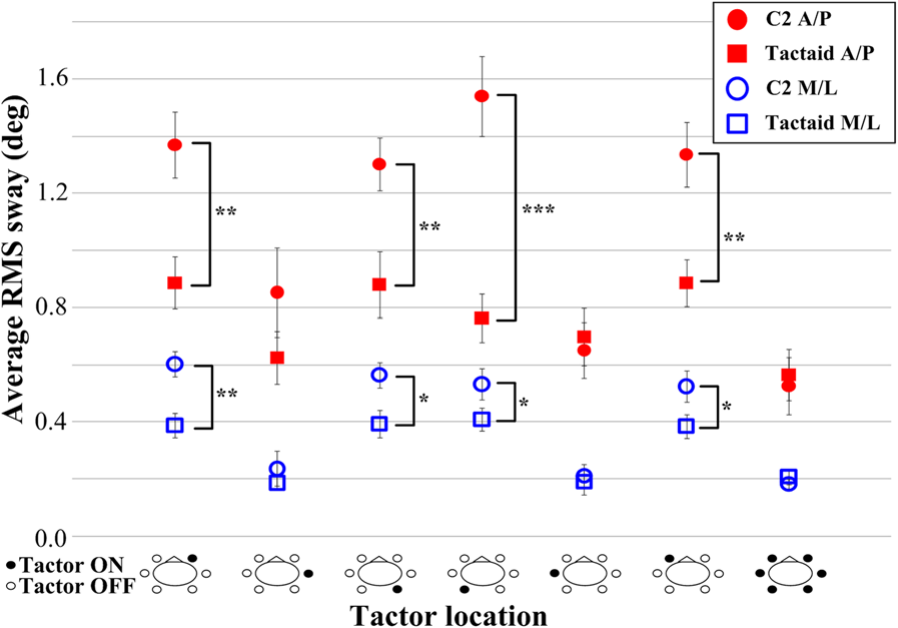
**Average A/P and M/L RMS sway values for the C2 (circles) and Tactaid (squares) tactors during vibration as a function of tactor location.** Red and blue symbols represent the A/P and M/L RMS sway values, respectively. Error bars indicate standard error of the mean (*p < 0.05, **p < 0.01, ***p < 0.0001). Bird’s-eye view drawings illustrate vibration locations.

Comparisons of the average RMS sway during vibration for each tactor type as a function of tactor location are illustrated in Figure 6. Both the A/P and M/L RMS sway magnitudes were significantly greater with C2 tactors than Tactaid tactors when vibration was applied over the internal oblique and erector spinae locations. However, Figure 6 shows that the A/P and M/L RMS sway magnitudes were similar (*p* > 0.05) for the C2 and Tactaid tactors when vibration was applied over the external oblique locations or over all locations simultaneously.

### PSD

Table 1 shows that the ANOVA applied to the PSD magnitude indicated that the main effects of tactor type, location, and period, and the “tactor type × location” and “location × period” interactions were significant in both A/P and M/L directions. Post-hoc analysis found that both the A/P and M/L PSD magnitudes were significantly greater (Tactaid: *p* < 0.02 and C2: *p* < 0.001, Tukey HSD) during than before or after vibration when stimulation was applied over the internal oblique and erector spinae locations. There were no differences (*p* > 0.05) between the A/P and M/L PSD magnitudes during and after the vibration periods for the internal oblique and erector spinae locations; during, before, and after the vibration periods across the six single locations; and during vibration across the internal oblique and erector spinae locations regardless of tactor type.

**Figure 7:**
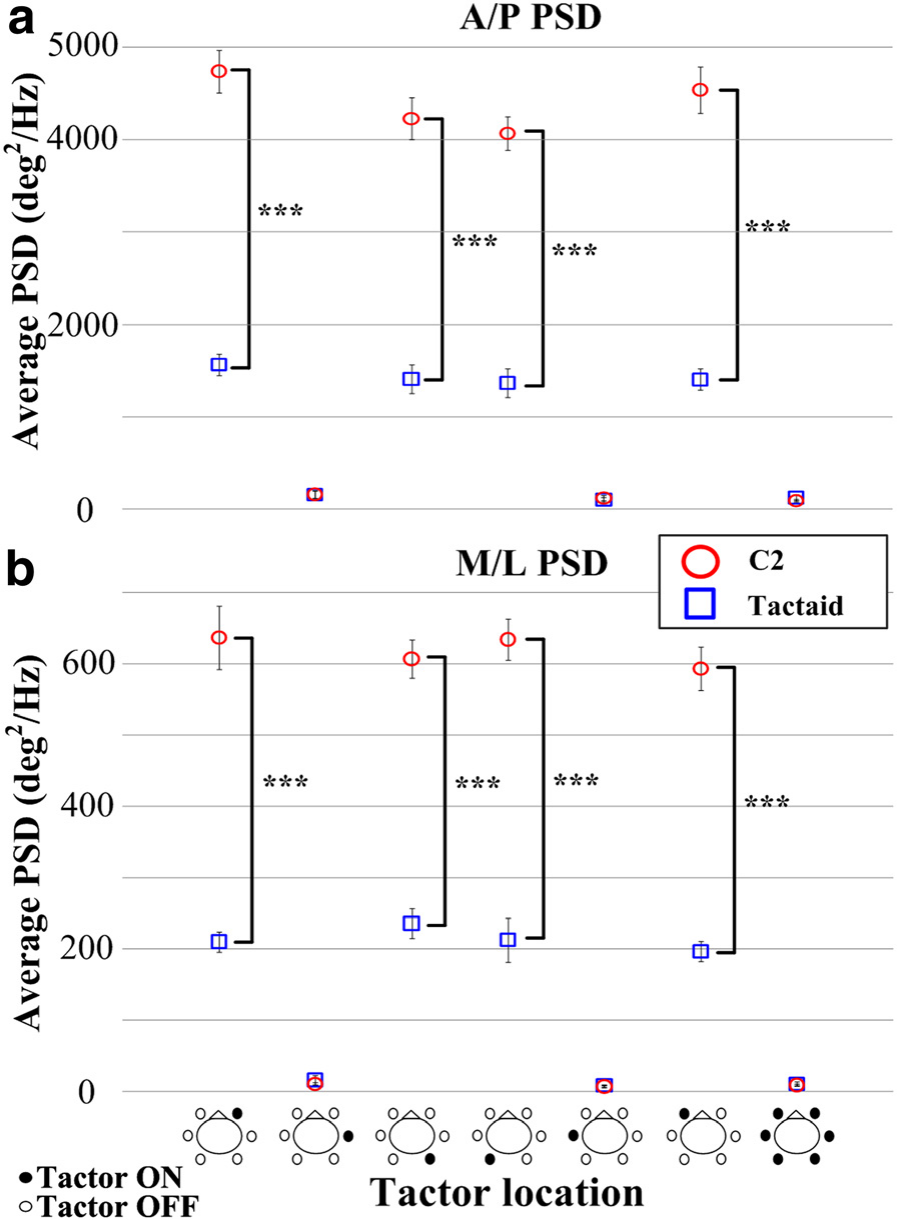
**Average A/P (a) and M/L (b) PSD magnitudes (frequencies less than 0.6 Hz) for the C2 (circles) and Tactaid (squares) tactors during vibration as a function of tactor location.** Error bars indicate standard error of the mean (***p < 0.0001). Bird’s-eye view drawings illustrate vibration locations. Note that the scale in (**a**) is ten times greater than that in (**b**).

Figure 7 compares the average PSD magnitudes during vibration for each tactor type as a function of tactor location. Both the A/P and M/L PSD magnitudes were significantly greater with C2 tactors than Tactaid tactors when vibration was applied over the internal oblique and erector spinae locations. However, there were no differences (*p* > 0.05) in A/P and M/L PSD magnitudes between the C2 and Tactaid tactors when vibration was applied over the external oblique locations or over all locations simultaneously.

### Subjective evaluation of the vibration strength

According to the post-test survey, all subjects reported that, for each tactor type, the magnitude of vibration “felt the same” across the locations. In addition, nine of eleven subjects indicated that the vibration intensity was higher for the C2 tactors than for the Tactaid tactors; one subject reported the opposite and one subject did not perceive a difference in vibration intensity.

## Discussion

The results show that the direction of vibration-induced postural shifts is a function of the selected stimulation location around the torso, while the magnitude of the postural shifts is a function of the tactor type used to generate the vibrotactile stimulus. A significant postural shift towards the location of the applied vibration was observed when stimulation was applied over the internal oblique and erector spinae muscle locations. The direction of the observed postural shift was not dependent on tactor type. These findings suggest that cutaneous information from the skin over the muscles of the torso contributes to the proprioceptive internal representation of the upper body and its orientation. Indeed, the directional shift was congruent with a postural response resulting from the lengthening of an abdominal muscle which is accompanied by skin stretch. Such responses also occur when vibration stimulates the muscle spindles [36,37]. Hence, the vibration-induced activity of cutaneous receptors is likely interpreted as a skin stretch corresponding to proprioceptive information, as shown for distal joints [20]. The latency of the postural response for stimulation over the internal oblique and erector spinae locations is substantially greater than that of a reflex response, which is known to be less than 100 ms [38,39]; thus, a significant role of reflex contribution and muscle proprioception to changes in posture is ruled out. This hypothesis, discussed in detail in our previous study [24], is briefly outlined here.

Vedel and Roll [40] and Ribot-Ciscar et al. [31] have shown that mechanoreceptors are very sensitive to mechanical vibration with stimulations in the range of 200–500 μm peak-to-peak displacement. The magnitude of both the postural shifts and the RMS sway occurring in response to an applied vibratory stimulus was significantly larger when the C2 tactor was employed versus the Tactaid tactor. This was to be expected, since the stimulation magnitude was approximately four times greater for the C2 tactors than for the Tactaid tactors. Furthermore, the subjects reported that the magnitude of the vibration across the locations was perceived to be the same for a given tactor type. Although it could not be experimentally controlled, given the subjective responses of the participants we assumed that the tactor contact pressure was fairly equally distributed around the torso by the elastic belt. However, the subjects indicated that the perceived vibration intensity (i.e., displacement amplitude) was greater for the C2 tactors than for the Tactaid tactors. This difference in perception is in agreement with the difference in postural responses and is well correlated with vibration strength. Furthermore, this finding is in agreement with investigations by Martin et al. [41], who show that the strength of vibration-induced proprioceptive activity increases with the magnitude of the vibration stimulus. Kavounoudias et al. [36] and Wierzbicka et al. [42] have also shown that the postural responses induced by vibration of the ankle muscles increase with stimulation magnitude. Therefore, we assume that due to the greater strength of the C2 tactor, a larger number of tactile receptors are recruited by C2 than Tactaid stimulation, which in turn increases the associated compensatory response. The efficiency of the stimulation may also be greater for linear tactors, such as the C2, than for inertial actuators, such as the Tactaid, since the C2 may produce a larger deformation of the skin due to the unique direction of travel of the generated pulse waves. Hence, a better efficiency may be obtained by a more secure driving of the cutaneous receptors. In other words, the consistency of receptor response to each vibration cycle would be greater for normal stretch than for shear stretch.

The drifts of postural responses are monotonous and reach a peak at approximately the same time for both tactor types; however the peak is greater for the C2 tactors than for the Tactaid tactors. One possible interpretation is that more secure driving and a larger recruitment of tactile receptors increase response speed, as indicated by the results related to vibration-induced illusions, since the speed of vibration induced illusory movements [37,43,44] or real movements [37] is in proportion to frequency. Furthermore, the average value of the PSD mean power frequency (frequencies less than 0.6 Hz for both A/P and M/L directions) was not significantly different in the presence or absence of vibrotactile stimulation regardless of the tactor type or tactor location. Since the measured postural sway frequency lies within the normal range of less than 1.0 Hz [13,45], vibrotactile stimulation does not appear to induce a disruptive increase in sway frequency, but rather an adjustment of posture associated with proprioceptive information.

Vibration applied to the skin over the external oblique muscle locations did not induce a significant shift regardless of the tactor type. Indeed, postural stability is usually greater in the M/L than A/P direction during normal stance [41] and, in the present study, the hip-width separation of the feet also contributed to a high lateral stability. Hence, a small vibration-induced change in sensory information is less likely to induce a compensatory postural response in the direction corresponding to the action of these muscles, since stability may not be perceived to be compromised.

The results of the present study show a vibration-induced inclination of the torso; however, the measurements of postural trajectories at the torso level do not allow for the description of a possible reorganization of posture implicating a multi-segmental response (e.g., head, upper body, lower body). Thus, further investigation is necessary to assess the relative contribution among different body segments (i.e., the reorganization of different body segments for postural coordination).

Our experimental findings suggest that tactor type and application locations should be carefully considered when designing vibrotactile displays to be used around the torso. Moreover, the choice of instructions concerning corrective movements requires additional investigation to determine their compatibility with the non-volitional response to the vibrotactile stimulation. It has yet to be determined whether or not the use of attractive instructional cues (“move in the direction of the vibrotactile stimulus”) facilitates a postural response during vibrotactile biofeedback balance applications. The instructional cue may change the cognitive interpretation of the cutaneous information generated by the vibration and thus the compatibility of the response direction with that stimulation.

## Conclusion

This study describes induced postural shifts in the direction of the vibration location when stimulation is applied to the skin over the internal oblique and erector spinae muscle locations regardless of tactor type. The compensatory motor response to the stimulation of cutaneous receptors corresponds to an attraction in the direction of the stimulated area. These findings suggest that attractive instructional cues be considered when designing torso-based vibrotactile displays for balance applications.

## Abbreviations

ANOVA: Analysis of variance; A/P: Anterior-posterior; DFT: Discrete Fourier transform; IMU: Inertial measurement unit; M/L: Medial-lateral; PSD: Power spectral density; RMS: Root-mean-square

## Competing interests

The authors declare that they have no competing interests.

## Authors’ contributions

B-C L participated in the design of the study, developed the software required to run the experimental instrumentation, collected the subject data, conducted the data and statistical analysis, and helped to draft the manuscript. B M participated in the design of the study, co-defined the data analysis methodologies, interpreted the results, and helped to draft the manuscript. K S conceived of the study, supervised the research, co-defined the data analysis methodologies, and helped to draft the manuscript. All authors read and approved the final manuscript.
